# From Resilience to Vulnerability: Mechanistic Insights into the Effects of Stress on Transitions in Critical Period Plasticity

**DOI:** 10.3389/fpsyt.2013.00090

**Published:** 2013-08-13

**Authors:** Bridget L. Callaghan, Bronwyn M. Graham, Stella Li, Rick Richardson

**Affiliations:** ^1^School of Psychology, The University of New South Wales, Sydney, NSW, Australia

**Keywords:** maternal-separation, FGF2, fear conditioning, memory retention, extinction, development, infant, critical period

## Abstract

While early experiences are proposed to be important for the emergence of anxiety and other mental health problems, there is little empirical research examining the impact of such experiences on the development of emotional learning. Of the research that has been performed in this area, however, a complex picture has emerged in which the maturation of emotion circuits is influenced by the early experiences of the animal. For example, under typical laboratory rearing conditions infant rats rapidly forget learned fear associations (*infantile amnesia*) and express a form of extinction learning which is relapse-resistant (i.e., extinction in infant rats may be due to fear erasure). In contrast, adult rats exhibit very long-lasting memories of past learned fear associations, and express a form of extinction learning that is relapse-prone (i.e., the fear returns in a number of situations). However, when rats are reared under stressful conditions then they exhibit adult-like fear retention and extinction behaviors at an earlier stage of development (i.e., good retention of learned fear and relapse-prone extinction learning). In other words, under typical rearing conditions infant rats appear to be protected from exhibiting anxiety whereas after adverse rearing fear learning appears to make those infants more vulnerable to the later development of anxiety. While the effects of different experiences on infant rats’ fear retention and extinction are becoming better documented, the mechanisms which mediate the early transition seen following stress remain unclear. Here we suggest that rearing stress may lead to an early maturation of the molecular and cellular signals shown to be involved in the closure of critical period plasticity in sensory modalities (e.g., maturation of GABAergic neurons, development of perineuronal nets), and speculate that these signals could be manipulated in adulthood to reopen infant forms of emotional learning (i.e., those that favor resilience).

## Introduction

Early life experiences have long been considered critical for the establishment of mental health. Exposure to a range of childhood adversities such as maladaptive family functioning, rearing in an institutional setting, and trauma lead to increased mental health risk and difficulties in emotional regulation and cognitive functioning (1–5). In both humans and non-human species the early rearing environment has been shown to influence the development of brain regions critical to emotional processing and/or mental health outcomes (3, 6–8). Despite the recognized importance of early life experiences in the establishment of mental health however, there has been surprisingly little empirical research which examines the role of early experiences (such as adverse rearing) on the development of emotional learning. Yet some forms of emotional learning (e.g., learning to fear and learning to inhibit fear responses) are critically involved in both the establishment and treatment of mental health disorders in humans [see (9), for a review (10–12)]. Further, evidence from animal models has demonstrated considerable developmental heterogeneity in the processes involved in fear learning and fear inhibition (13–18). Hence, understanding the maturation of emotional learning and how its developmental trajectory is altered by different early experiences might aide in our understanding and treatment of mental health disorders across the lifespan.

In this review we describe the normal trajectory of fear learning across the infancy to juvenile periods of development in the rodent and discuss how developmental dissociations in these learning processes are altered by a variety of early life experiences (specifically, exposure to early life adversity or fibroblast growth factor-2; FGF2). Considering the high degree of similarity in fear learning outcomes following early manipulation of the rearing environment and FGF2, we propose a model via which the experience of early adversity might activate, within the limbic circuit, molecular signals known to be involved in critical periods of plasticity in other brain regions via an FGF2-dependent pathway. The review ends with a discussion on how the proposed model might guide further pre-clinical research in this field as well as highlighting potential areas for translation to humans.

## Developmental Differences in Fear Learning

In recent years, studies using Pavlovian fear conditioning have demonstrated a number of fundamental differences in emotional learning in infant and adult animals. During a typical Pavlovian fear conditioning procedure an initially neutral conditioned stimulus (CS; e.g., noise) is paired with an aversive unconditioned stimulus (US; e.g., footshock). Such pairings rapidly lead the animal to express a species-specific defensive/fear response toward the CS [e.g., freezing in the rat; (19)]. Although both infant and adult rodents can learn a CS-US association during fear conditioning, their retention of those fear memories differs dramatically. Specifically, following fear conditioning adult rats will typically express fear to that cue for the rest of their life (20). Infant rats, on the other hand, exhibit rapid forgetting, a phenomenon known as *infantile amnesia* (13). For example, when given two pairings of a white noise CS with a foot shock US, both infant [i.e., postnatal day (P) 16] rats and juvenile (i.e., P23) rats show equivalent levels of fear immediately after training (18). However, when tested 2 days after training, infants show a dramatic decrease in fear, while juveniles continue to express a high level of fear in the presence of the CS. This suggests that while infant animals can acquire fear just as readily as older animals, they do not retain the memory across an extended period of time (13, 21, 22). This profound and spontaneous forgetting is not limited to infant rats but is experienced by all altricial animals, including humans (23). For example, humans are generally unable to recall events that occurred prior to the age of 3 years and have hazy memories of events that occurred until around 5–6 years of age (24).

One question of interest to neuroscience researchers is what happens to the memory trace following infantile amnesia. That is, does the forgetting represent decay in the memory trace, leading to eventual erasure of that memory, or are infant memories simply unable to be retrieved? The evidence suggests that infantile forgetting often represents a retrieval failure. Numerous studies have shown that a pre-test reminder treatment effectively reverses the deficit in retention, suggesting that infantile amnesia is caused by a failure of cues to spontaneously retrieve the memory trace (15, 25–27). In addition, reducing GABAergic inhibition in the infant rat at test (via systemic injection of FG7142; a partial inverse agonist of the GABA_A_ receptor) leads to a forgotten memory being expressed (15, 28). Interestingly, studies have shown that administration of midazolam, which increases GABA_A_ activity, in adult rats has strong amnestic effects (29), suggesting that infantile forgetting may be an exaggerated form of adult memory loss.

## Developmental Differences in Fear Inhibition

Another area where developmental differences are observed is in the inhibition of fear. That is, once fear is acquired it can then be decreased or inhibited through a process known as extinction. During a typical extinction procedure the animal is repeatedly exposed to the CS without the reinforcing US (e.g., shock). In the last decade, extensive research has been conducted examining the behavioral, neural, and molecular mechanisms underlying fear extinction. On a behavioral level, it is widely accepted that extinction in older animals (e.g., juvenile and adult animals) is not simply erasure of the original fear memory. Instead, extinction is believed to involve the formation of a new inhibitory (CS-noUS) memory. Evidence for the “new inhibitory learning” account of extinction comes from both rodent and human studies showing that fear can return following extinction training through either a change in context [renewal; e.g., (30, 31)], presentation of an aversive stimulus [reinstatement; e.g., (32, 33)], or simply the passage of time [spontaneous recovery; e.g., (33, 34)]. Thus, in older animals, extinction is relapse-prone.

The idea that extinction involves new learning in juvenile and adult animals is further supported by evidence from pharmacological studies demonstrating that extinction involves the same cellular mechanisms as other forms of new learning. For instance, both fear conditioning and fear extinction require activation of the *N*-methyl-d-aspartate receptor (NMDAr), as administration of dl-2-amino-5-phosphonovaleric acid (APV; an NMDAr antagonist) either systemically or directly into the brain disrupts both forms of learning (35–37). Conversely, systemic or intra-amygdala administration of the NMDAr partial agonist d-cycloserine (DCS) enhances extinction retention (38–40). Other cellular mechanisms involved in the mature form of fear extinction have also been explored. For instance, along with NMDAr transmission, fear extinction in juvenile/adult rats has been shown to rely on GABAergic (41, 42) and opioidergic transmission (43, 44).

The characteristics of extinction in infant rodents have also begun to be explored and the results suggest that infant rodents exhibit a qualitatively different extinction profile compared to juvenile and adults. Whereas adult animals exhibit relapse-prone extinction, infants exhibit *relapse-resistant* fear extinction. That is, infant P17 animals do not show renewal, reinstatement, or spontaneous recovery following extinction (16, 42, 45, 46). The lack of relapse behavior seen after extinction in the young animal suggests that extinction at this age is mediated by a fundamentally different mechanism, which might be best characterized as erasure of the original fear memory rather than new learning. In support of this possibility, other studies have demonstrated that extinction in infant animals is not dependent on NMDArs (47); in contrast to P24 rats, systemic administration of the NMDAr antagonist MK-801 did not impair extinction retention in P17 animals. This effect is not due to a generalized lack of NMDAr-involvement in infant learning because the same drug was shown to impair fear acquisition in rats when given prior to conditioning in infancy. These findings suggest that while NMDArs are involved in some forms of learning during infancy (i.e., fear conditioning), they are not involved in others (i.e., fear extinction).

Other neurotransmitters have also been shown to differentially modulate early extinction memories. For instance, unlike juvenile and adult rats, GABAergic transmission does not affect long-term extinction in infant rats (42), suggesting that extinction does not involve formation of a new “inhibitory” association in young rats. On the other hand, some neurotransmitter systems do appear to be involved in extinction across age. Specifically, endogenous opioids appear to regulate extinction in infant animals, as P17 rats given the opioid receptor antagonist naloxone exhibited impaired within-session extinction compared to animals given saline (44); a finding which is similar to that seen in adult rats (43).

The developmental differences in fear inhibition are not only observed on the behavioral and pharmacological levels as there are also marked differences in the neural circuitry which supports extinction across development. In adult animals, lesion, immunohistochemical, and electrophysiological studies have implicated the amygdala, medial prefrontal cortex (mPFC), and hippocampus in the extinction of fear [see (48–50), for extensive reviews on the role of these structures in extinction]. Specifically, a widely accepted neural model of extinction proposes that the amygdala is involved in the acquisition and consolidation of learned fear [e.g., (51)], while the mPFC is important for regulating the expression of fear through either inhibiting or exciting amygdalar neuron output [e.g., (50)]. Additionally, the hippocampus appears to be involved in the contextual modulation of extinction through its projections to the mPFC (52, 53).

While this neural model of extinction has been predominantly based on rodent studies, there is evidence to suggest that a similar circuitry is involved in regulating emotional memories in humans (54). For example, Phelps and colleagues showed that the mPFC-amygdala circuit is activated in humans following extinction training (55), while Kalisch et al. (56) found that retrieval of a context-dependent extinction memory activated the hippocampal-mPFC circuit. Interestingly, this “extinction circuit” has been shown to be dysfunctional in individuals with post-traumatic stress disorder (PTSD). Specifically, some studies have found that individuals with PTSD exhibit *hypoactivation* of the fear inhibition components of the circuit (i.e., mPFC and hippocampus) and *hyperactivation* of the fear activation components of the circuit (i.e., amygdala), relative to healthy controls [e.g., (12, 57)].

While the extinction circuit has been well documented in adult rodents and adult humans, until very recently this circuit had not been examined at earlier stages of development. Over the past 5 years, however, some progress has been made in mapping the neural circuitry mediating extinction in the developing animal. Those studies indicate that if extinction occurs in the juvenile stage of development, then it involves the same neural circuit as extinction in adulthood. In contrast, extinction in the infant stage of development appears to involve a different circuit. For example, Kim and Richardson (58) found that inactivating the amygdala (via infusion of the GABA_A_ agonist muscimol) prior to extinction significantly impaired long-term extinction in both P24 and P17 rats. Further, it was observed that there was an increase in the number of phosphorylated mitogen-activated protein kinase (pMAPK) neurons in the basolateral amygdala (BLA) following extinction training in rats of both ages (59). Therefore, it seems that the amygdala is an important structure for the extinction of conditioned fear in rats, regardless of age. In contrast, the mPFC appears to mediate fear extinction only in older animals [i.e., juveniles and adults; (59)]. In that study, infusion of muscimol into the mPFC prior to extinction training impaired extinction retention in P24 rats but not in P17 rats. In addition, while extinction training caused an increase in pMAPK-labeled neurons in the mPFC of P24 rats, there was no extinction-related change in pMAPK-labeled neurons in that structure in younger animals. Together, the research on fear extinction in the infant rat appears to suggest that infants recruit a much simpler neural circuit during extinction than do rats extinguished at later stages of development (i.e., juvenile through to adulthood). It has been proposed that these neural differences in extinction might underlie the less flexible extinction behavior seen in infant rats. That is, perhaps the lack of relapse following infant extinction in the rat is the outcome of a simple extinction circuit which cannot integrate multiple, contextually gated associations. See Table 1 for a summary of the behavioral and neural differences in extinction and fear retention across development.

**Table 1:** **Summary of the behavioral and neural characteristics of the fear retention and extinction systems in adult and infant (<P21) rodents**.

	Adult rodent	Infant rodent	Infant rodents following early stress/CORT/FGF2
Renewal	✓	×	✓
Reinstatement	✓	×	✓
NMDA	✓	×	?
GABA	✓	×	✓
Endogenous opioids	✓	✓	?
Amygdala	✓	✓	?
mPFC	✓	×	?
Good fear retention	✓	×	✓

*✓Indicates that the phenomenon is present in age or treatment groups; × indicates that the phenomenon is absent in age or treatment groups; ? indicates that the phenomenon has not yet been examined in the age or treatment group. See text for definition of the terms used in this table*.

The current literature clearly indicates that fear retention and fear inhibition are dynamic processes that exhibit considerable developmental heterogeneity. Whereas infant rats exhibit marked forgetting and use a simpler extinction system characterized by a resistance to relapse, older rats demonstrate better memory retention and use a more flexible neural circuit that results in relapse-prone extinction learning. While examination of these differences has occurred primarily in animal models, there is evidence that at least one of the transitions (i.e., the transition from infantile amnesia to adult-like memory retention) also occurs in developing humans. It is now commonly accepted that memories formed before the age of approximately 3 years in humans are generally inaccessible to conscious recollection in adulthood [e.g., (24, 60)]. While much of the human research on infantile amnesia has focused on various cognitive factors that might contribute to the effect [e.g., language acquisition, development of self-concept, increasing ability to utilize reminder cues; (61–63)], the occurrence of the same effect in non-human animals suggests that more basic neurobiological mechanisms might provide a better account for infantile amnesia. In contrast to the complimentary findings on infantile amnesia across rodents and humans, there hasn’t been any, to our knowledge, research examining whether the transition from relapse-resistant to relapse-prone extinction is also a feature of human development. Future studies should examine whether the transition in extinction mechanisms also occurs in humans.

The fact that developmental transitions in emotional learning take place in humans as well as rodents is of particular interest, suggesting that findings in either species might be successfully translated to the other. Indeed, a mechanistic understanding of the developmental transitions in emotional learning across species might have considerable clinical implications because anxiety disorders are characterized by persistent expression of fear and high rates of treatment relapse. In an effort to uncover some of the mechanisms which regulate the expression of infant fear learning within a rodent model, some very recent studies have begun to examine factors which are involved in the transition from infant- to adult-like fear learning, with a view to manipulating these mechanisms in adulthood to promote infant-like forgetting and relapse-resistant extinction.

## Early Experiences Regulate the Transition between Infant- and Adult-Like Fear Learning in Rodent Models

Two different types of early experience have recently been shown to affect the age at which rats transition between infant- and adult-like fear learning. While these experiences are vastly different in nature, they both appear to impact the developmental transition in fear learning in similar ways (i.e., both manipulations lead to early expression of adult-like fear retention and extinction behaviors).

### Stress

It has been known for decades that exposure to stressors or stress hormones (corticosterone; CORT) can program the maturation of fear responding. For example, rats begin to exhibit species-specific defense responses (freezing, inhibition of ultrasonic vocalizations; USV) to the presence of a strange adult male/male odor at approximately P10. Further, while the amygdala is not activated by the presentation of a male odor in rats younger than P10, amygdala activation is increased following presentation of the same stimulus in rats aged P10 and older (64–66). Defense responding and amygdala activation can be elicited by presentation of a potential predator odor earlier if rats are given exogenous CORT at P8. Further, these responses can be delayed if rats are adrenalectomized, which leads to a reduction in CORT [i.e., removal of the adrenal gland and subsequent reduction in CORT; (66–69)].

In addition to the stress-induced acceleration of unlearned fear reaction development, the maturation of learned fear reactions also appears to be affected by stress exposure. For example, in the second postnatal week of life rats exhibit a developmental transition in their behavioral and neural response to an odor previously paired with shock. Specifically, in rats aged P10 and older odor-shock conditioning leads to subsequent avoidance of the shock-paired odor and activation of the amygdala. However, rats conditioned at P6–P8 exhibit a paradoxical approach response toward the odor (70, 71). In addition, presentation of the shock-paired odor does not lead to increased activity in the amygdala of P8 rats (72), suggesting that different neural structures are involved in the conditioned responses exhibited by P10 and P8 rats. Interestingly, if rats were raised in a stressful rearing environment, or were given a CORT injection before test, then a precocious avoidance response to the shock-paired odor was observed at P8, which was correlated with increased amygdala activity (72–75). Thus, early life stress in rodents accelerates the transition between infant- and adult-like behaviors and neural responses in odor-shock associative learning just like it accelerates the development of unlearned fear responses to a potential predator odor.

Although environmental effects on the maturation of fear responses have been investigated for some time, how the environment affects development of fear retention and fear extinction has only recently begun to be investigated. Interestingly, those studies show that early exposure to stress or CORT also accelerates the maturation of fear retention and extinction learning. Specifically, compared to a group of standard-reared (SR) infant rats, infants exposed to maternal-separation (MS; 180 min separation from P2 to P14) before conditioning on P17 express fear memories for longer periods of time (76). While SR infants forgot a conditioned association in as little as 10 days, MS infants expressed memory for the conditioned association up to 30 days after training. Similarly, pups that were suckled by a SR mother that had been exposed to CORT in her drinking water (from P2 to P14), but not pups suckled by vehicle-exposed mothers, also exhibited longer retention of fear memories. Taken together, those results suggest that early stress/CORT exposure leads to an accelerated transition in the fear retention system used by infant rats. In other words, rats make a precocious transition from the infantile amnesia system to the adult-like retention system following exposure to stress/CORT.

It is not only an early transition into adult-like retention that is seen following MS however. In another set of studies the effect of MS on the expression of two relapse phenomena after extinction (fear renewal and reinstatement) was examined in infant rats (77). It was shown that while the SR infant rats did not exhibit either of those relapse phenomena [replicating past findings in P17 rats; (16, 45)], the MS infants did. In other words, following MS rats made an early transition from the infant relapse-resistant extinction system to the adult-like relapse-prone extinction system. In addition to exhibiting increased relapse, the expression of extinction in MS P17 rats was also found to be dependent on activation of GABA_A_ receptors. As mentioned earlier, the expression of adult extinction memories requires activation of the GABA_A_ receptors (41). Similar to studies in adults, when GABAergic inhibition was decreased at an extinction test in juvenile rats (via injection of FG7142), extinction retention was impaired (42). However, in that study FG7142 had no effect on levels of expressed fear in infant rats. That is, infant rats exhibited low levels of freezing at test following extinction regardless of whether they received FG7142 or not. Interestingly, when MS infant rats were given FG7142 at test they behaved similarly to juvenile and adult rats, suggesting that after early stress the role of GABA_A_ receptors in extinction expression becomes more adult-like (77). These studies suggest that the development of fear retention and extinction learning, two behaviors with potential importance for vulnerability to mental health disorders (e.g., PTSD), are dynamically regulated by the early life rearing environment (see Table 1 for a summary) and that stress is one condition under which increased vulnerability to mental health problems might emerge.

### FGF2

Another early life event that has been shown to influence the development of fear learning and extinction is exposure to fibroblast growth factor-2 (FGF2). FGF2 is a neurotrophic factor that regulates cell proliferation, differentiation, and survival. During early development FGF2 is responsible for determining the overall morphology of the brain, and during adulthood it is released in response to stress or brain injury, potentially playing a neuroprotective role (78, 79). Early life exposure to FGF2 has marked central effects; a single peripheral administration of FGF2 on P1 led to increased cell proliferation in the hippocampus, resulting in a larger hippocampal volume that was first evident at P4 and that persisted throughout adulthood (80). Conversely, transgenic mice that lack FGF Receptor 1 (the primary receptor for FGF2) have decreased hippocampal cell proliferation, resulting in permanent hippocampal atrophy (80, 81).

Graham and Richardson (82) investigated whether these long-term hippocampal morphological changes induced by early life exposure to FGF2 might lead to changes in hippocampal-mediated memory formation. They first examined the impact of early life FGF2 on contextual fear conditioning in the developing rat. Infant rats exhibit impaired long-term (i.e., after 24 h) memory for contextual fear relative to older rats (83). However, subcutaneous injections of FGF2 from P1-5 led to an early emergence of long-term memory for contextual fear in P16 rats. Early life FGF2 also enhanced contextual fear conditioning in P23 rats, an age at which rats exhibit moderate levels of long-term memory for contextual fear.

Graham and Richardson (82) then examined the impact of early life FGF2 on fear extinction at P16. In those studies, cued fear conditioning procedures were used (i.e., white noise CS paired with shock US) as infant rats can exhibit long-term memory of such associations. Animals were trained in one context, and then extinguished in a different context. Early life FGF2 did not affect the strength of cued fear conditioning, the rate of extinction acquisition, or the retention of extinction training when the extinguished CS was presented in the extinction training context. However, when the extinguished CS was presented in the original fear conditioning context, FGF2-treated P16 rats exhibited recovered fear responses whereas vehicle-treated P16 rats exhibited low fear responses. That is, early life FGF2 led to a precocious emergence of renewal. These results show that early exposure to FGF2 causes an accelerated emergence of the ability to encode and/or maintain a representation of the contextual elements associated with fear conditioning and extinction memories. When taken together with the findings from Cheng et al. (80) it is possible that these behavioral results are a consequence of the effects of early life FGF2 on hippocampal development.

The fact that FGF2, maternal-separation, and exposure to CORT have similar effects, all accelerating the development of fear learning in infant rats, raises the possibility that stress and FGF2 produce their outcomes on early fear learning and extinction through the same or a similar pathway. For example, it might be the case that FGF2 is one of the mechanisms involved in accelerated maturation following early stress. In support of this idea, a large body of evidence has suggested that FGF2 is critically involved in the effects of stress. FGF2 appears to be modulated by activation of the hypothalamic-pituitary-adrenal (HPA) axis, which mediates the mammalian response to stress. Adrenalectomized rats exhibit reduced expression of FGF2 in the hippocampus, striatum, and frontal cortex, whereas administration of glucocorticoids increases FGF2 mRNA in the hippocampus and prefrontal cortex; both results support the idea that adrenal hormones (which are responsible for terminating the stress response) exert control over FGF2 [see review by (84)]. Indeed, both physical and psychological stress upregulate FGF2. Specifically, brain injury leads to increases in FGF2 around the site of the lesion, and application of FGF2 to the lesion reduces cell death and increases astrocytic density (85, 86). Likewise, restraint stress (a psychological stressor) increases FGF2 mRNA expression in the hippocampus and prefrontal cortex (84). These findings point to a potential neuroprotective role for FGF2 in response to stress [see (79)].

There are several factors that determine whether or not FGF2 increases in response to stress, one of which is the controllability of the stressor. Bland et al. (87) exposed two groups of rats to a series of tail shocks. One group could terminate the shock by turning a wheel; the other group were yoked to the first and could not control the shock, but experienced the same number and intensity of shocks as the first group. Escapable, but not inescapable, shock led to a significant increase in hippocampal FGF2 protein expression 2 h post-shock, and this effect persisted for 24 h. Furthermore, inescapable shock, but not escapable shock, led to a significant decrease in the proliferation of hippocampal neural progenitor cells. A later study demonstrated that escapable shock, but not inescapable shock, also causes increases in FGF2 mRNA expression in the PFC (88). Similarly, Turner et al. (89) reported that chronic (4 days) social defeat stress, in which a rat is exposed to an aggressive male rat of a different strain, down-regulates hippocampal FGF2 mRNA expression. These findings suggest that endogenous FGF2 may protect against the harmful effects of stress (perhaps by increasing cell proliferation), but only if the animal has some level of control over the stressor.

Another factor that determines FGF2’s involvement in the stress response is prior exposure to stress hormones. It has been shown that prenatal exposure to corticosterone significantly reduces basal FGF2 mRNA expression during adulthood. Furthermore, prenatal exposure to corticosterone significantly attenuates the upregulation of hippocampal FGF2 mRNA normally seen following acute stress in adulthood (84). Therefore it is possible that early life stress may alter (i.e., cause dysfunctions in) FGF2’s neuroprotective response to stress later in life (79).

## How Do Early Stress and FGF2 Exposure Accelerate the Development of Fear Learning Systems?

One intriguing possibility concerning the effects of stress and FGF2 exposure on accelerated emotional development is that these early experiences regulate the expression of critical period plasticity. Specifically, it is possible that infantile amnesia, impaired context learning, and relapse-resistant extinction represent forms of critical period plasticity in emotional systems, and that these forms of plasticity are controlled by the same cascade of signals as critical periods in other areas of the brain. That is, stress exposure could initiate a cascade of cellular and molecular changes involved in terminating infant-like forms of fear learning via HPA activation of FGF2 receptors. This would be an attractive, and simple, explanation for the similar outcomes of early stress and FGF2 exposure on developmental transitions in fear learning. In other words, it is possible that stress and FGF2 activate a “signature” set of signals involved in critical period termination across the brain.

Traditionally, critical/sensitive periods have been defined as discrete stages of rapid neural development in which plasticity is enhanced, allowing early environmental input to fine-tune final wiring patterns in the brain before plasticity is reduced in adulthood [e.g., (90–92)]. The onset and offset of critical periods is not a simple age-dependent maturational process. Rather, the timing of critical periods can be manipulated by different experiences which affect the various molecular and cellular signals involved in their opening and closure (90). While the high levels of plasticity inherent in a critical period allow for enhanced learning and refinement of neural functions these periods also represent a time of vulnerability for the developing brain. If aberrant sensory or social events are experienced, or expected environments do not manifest, then the timing and function of the critical period can be altered, placing the brain at risk for abnormal wiring patterns and adverse behavioral/sensory outcomes. For instance, some developmental disorders in humans (e.g., autism) have been proposed to result from a disruption in the timing or expression of critical periods across various brain regions (93, 94).

There are many different critical periods which occur across development, each involving unique brain regions or neural circuits (95). For example, critical periods in humans have been proposed for the development of sensory/sensory-motor, cognitive, and emotion systems [e.g., (4, 7, 96, 97)]. For instance, when learning takes place before the age of 7 years, acquisition of a second language usually occurs to a level that is grammatically indistinguishable from that of native speakers. However, mastery of a second language becomes progressively harder from 8 years onward (98, 99). Other research has shown that children need to be exposed to appropriate levels of cognitive, tactile, and emotional stimulation early in life in order to develop adequate cognitive functions and emotion regulation skills. Children reared in institutional settings which lack the appropriate levels of stimulation exhibit profound deficits in cognitive and emotional development, effects which are often permanent if children are not adopted before the age of 2 years [see (3), for a review; (100)].

In non-human animals critical/sensitive periods have also been shown to occur in a variety of sensory and emotional systems, such as song learning in birds, attachment learning in rats, and cortical responses to vibrissa stimulation in rats [see (101–103), for a review; (71, 104, 105)]. The best characterized animal model of critical period plasticity, however, is that of ocular dominance (OD) plasticity induced by monocular deprivation [(106, 107); see (91, 108), for reviews; see also (92), for a review]. Only during the critical period for OD plasticity does closure of one eye result in a loss of visual acuity in the closed eye (amblyopia) and a shift in the responsiveness of neurons in the primary visual cortex away from the closed eye.

Research investigating OD plasticity has highlighted numerous molecular and cellular signals which are involved in opening and closing this critical period. Importantly, these signals have been shown to regulate the timing of sensitive periods in other sensory modalities (104, 105), suggesting that there may be a general neural signature which guides critical period timing across the brain. Although the neural signature for critical period timing has mostly been investigated in sensory systems, recent evidence suggests that the same signals may also regulate sensitive periods in fear learning (46). Further, there is some evidence to suggest that those neural signals are regulated by particular types of early experience, suggesting a potential mechanism via which stress/CORT/FGF2 may have affected the timing of adult-like fear retention, context learning, and extinction described earlier. While a detailed analysis of the molecular and cellular events involved in triggering the onset and offset of critical period plasticity in the visual cortex is beyond the scope of this review [interested readers are referred to excellent past reviews on the topic: (91, 92, 108)], we provide a brief summary of those molecular and cellular signals important for critical period plasticity in the visual system that also may have a role in fear and extinction learning and that appear to be regulated by stress/CORT/FGF2.

### Signals involved in the opening of critical period plasticity

The onset of OD plasticity appears to be triggered by a change in the balance of excitation and inhibition in the visual cortex, mostly as a result of developmental increases in inhibitory activity. For example, 4 days of monocular deprivation starting on P25–P27 induces OD plasticity in wild-type mice but not in mice with a genetic knockout (KO) of the GAD65 gene, which inhibits GABA release. However, critical period plasticity could be rescued in GAD65 KO mice if levels of inhibition were artificially increased during monocular deprivation via infusion of a benzodiazepine directly into the visual cortex (109). Benzodiazepines were also successful in precociously inducing OD plasticity when monocular deprivation was performed in pre-critical period mice [P15–P20; (90)]. It appears that the maturation of intra-cortical inhibition is regulated by brain derived neurotrophic growth factor (BDNF) because genetic over-expression of BDNF across postnatal development accelerated the maturation of parvalbumin positive (PV+) GABAergic interneurons in the visual cortex and resulted in a precocious critical period (110, 111). Together these studies suggest that the molecular machinery for enhanced plasticity is present early in life but that maturation in GABAergic circuitry (e.g., PV+ interneurons and GABAergic synapses) pushes inhibitory activity beyond a certain threshold to trigger the opening of the critical period.

### Signals involved in the closure of critical period plasticity

While intra-cortical inhibition appears to be sufficient for the initiation of critical period plasticity, there are several mechanisms that appear to be involved in critical period termination, many acting as “structural brakes” which limit plasticity. For example, critical periods appear to be regulated by the appearance of extracellular matrix proteins – perineuronal nets (PNNs) – around the dendrites, axons, and cell bodies of GABAergic neurons. PNNs are believed to limit critical period plasticity by increasing stability of synapses via inhibition of axonal growth and sprouting. Appearance of PNNs in various brain regions correlates with termination of critical period plasticity in several different sensory systems [e.g., (92, 104, 105, 108)], and recently appearance of PNNs in the amygdala was shown to correlate with the termination of infant-like, relapse-resistant extinction learning and the transition into adult-like, relapse-prone extinction learning (46). Interestingly, when PNNs in the visual cortex or amygdala of adult rats are degraded via chondroitinase ABC (chABC), then the critical periods for OD plasticity and erasure-like extinction, respectively, are reopened (46, 112). This research strongly suggests that erasure-like extinction represents a form of critical period plasticity occurring in emotion circuits in the brain, and that termination of this form of infant plasticity appears to be regulated by some of the same structural brakes as critical period plasticity in sensory systems.

In addition to the formation of PNNs, other developmental factors also appear to be involved in limiting structural plasticity in the visual cortex and terminating the critical period for OD plasticity. For example, maturation of myelin basic protein (MBP) in the visual cortex has been shown to correlate with termination of the critical period for OD plasticity (113), potentially through inhibiting mechanisms of structural remodeling necessary for plasticity (114). The myelin associated growth inhibitor Nogo-66 is known to limit axonal regeneration following CNS damage because antagonizing the Nogo-66 receptor (NgR) promotes axonal regeneration following spinal cord injury in the rat (115). Interestingly, when the NgR was genetically deleted in mice and monocular deprivation occurred post-sensitive period (i.e., at P45) the mutant mice exhibited OD plasticity whereas wild-type mice did not (113). Hence, it appears that adult mice retain the capacity for enhanced plasticity but that increased myelination in the visual cortex which occurs across development acts as a structural brake, limiting OD plasticity.

Another factor that has been implicated in the closure of the critical period for OD plasticity is calcium/cAMP response element binding protein (CREB)-mediated gene transcription. Evidence for the role of CREB activity in OD plasticity comes from studies which have shown that monocular deprivation during the critical period stimulates CREB-mediated gene transcription whereas post-critical period monocular deprivation has a less pronounced effect on CREB-mediated processes (116). Further, when CREB activity is enhanced in adult mice (through the use of a transgenic mouse line expressing VP16-CREB, which leads to constitutively active CREB across life), it has been shown that persistent OD plasticity can be induced in the visual cortex (117). Also, inhibiting upstream regulators of CREB (e.g., PKA) in cats decreases OD plasticity during the critical period (118).

It has been proposed that CREB is important in terminating critical periods because it regulates the activity of plasticity-modulating genes (92). Studies examining candidate CREB-mediated genes that might be involved in OD plasticity have focused on micro RNA (mir) 132 which has been implicated in neural plasticity (119). In a recent study, increasing mir132 expression in mice before monocular deprivation blocked OD plasticity during the critical period (120), suggesting that mir132 acts as a brake on plasticity.

### Stress, corticosterone, and FGF2 regulate molecular and cellular signals involved in critical period timing

**Figure 1:**
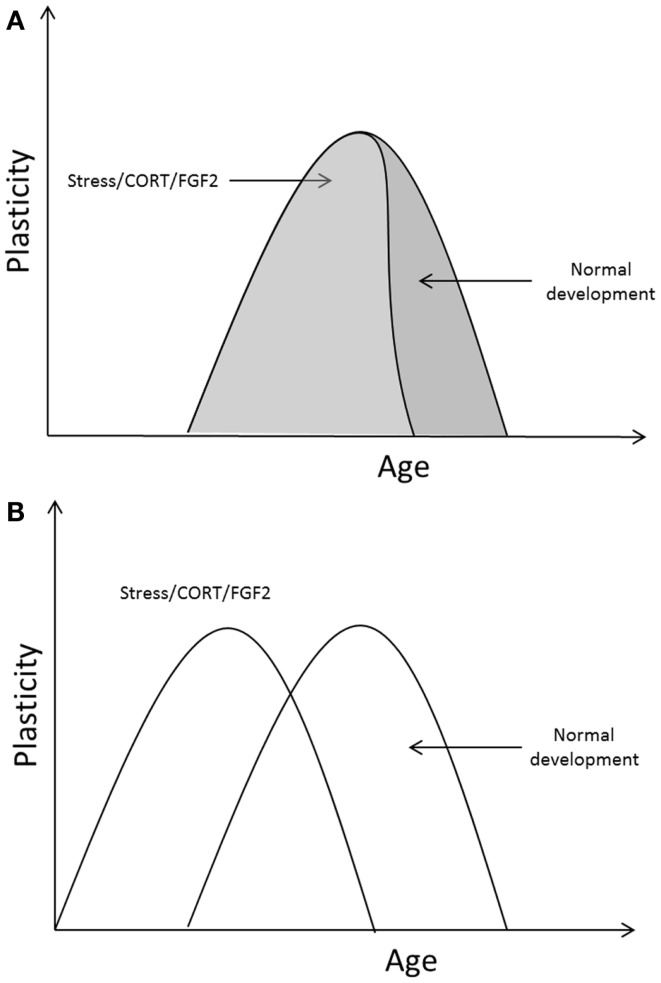
**Two potential outcomes of the effect of stress/CORT/FGF2 on critical period timing in the emotion system**. **(A)** Different manipulations may alter the duration of the critical period but may not affect the age at opening. **(B)** Once opened, the duration of the critical period may be relatively static; manipulations causing an early opening of the critical period in emotional plasticity would also cause an early closure.

Early exposure to stress, corticosterone, and FGF2 has been shown to accelerate the transition into adult-like fear retention and extinction learning in infant rats; early exposure to those events led to a precocious termination of the critical period for infantile amnesia and erasure-like extinction. It is possible that stress/CORT/FGF2 exposure hastened the developmental transitions in fear learning by acting on those processes known to be involved in critical period regulation in other systems. Indeed, evidence shows that early life adversity, CORT, and FGF2 regulate many of the molecular and cellular signals involved in both the opening and the closure of critical periods, accelerating the developmental emergence of those signals in brain regions important for emotional responding in adults. However, those rodent studies which examined environmental regulation of infant fear retention and extinction only measured outcomes at one time point making a determination of the early closure of the critical period possible but determination of an early opening of the critical period uncertain. It could be the case that the critical period for infantile amnesia and erasure-like extinction opened at the same time in MS/CORT/FGF2 and SR/vehicle rats, but that this period closed earlier in the MS/CORT/FGF2 rats (i.e., the time frame for the critical period was compressed). Alternatively, it may be the case that MS/CORT/FGF2 led to an early opening as well as an early closure of the critical period (see Figure 1 for a depiction of these possibilities). The fact that stress and FGF2 appear to regulate signals involved in both the opening and closure of critical periods, however, suggests that the latter case is most likely the case (i.e., that stress/FGF2 leads to an early opening and closure of the critical period in fear learning).

Evidence that stress might regulate critical period opening in the emotional system comes from studies examining the effect of early stress on GABAergic development. Specifically, maternal-separation has been shown to lead to a more mature form of GABAergic signaling in the CA1 region of the infant hippocampus of male rats (121), and maternally separated rats also exhibit a short-term upregulation of BDNF in the PFC and hippocampus at P17 (122). As mentioned earlier, the critical period for OD plasticity is triggered by maturation of GABA in the visual cortex. Further, early over-expression of BDNF in the visual cortex was shown to accelerate GABAergic maturation and lead to a precocious emergence of OD plasticity.

In addition to influencing signals involved in the opening of critical periods, early life stress/corticosterone/FGF2 also appear to regulate some of the structural brakes on plasticity. For example, early life stress (caused by weaning rats at P14 rather than P21) has been shown to accelerate whole-brain, as well as amygdala-specific, myelination in P21–P35 male mice (123, 124). Also, elevated glucocorticoids have been shown to accelerate the initiation and rate of myelination in co-cultures of Schwann-cell and neurons taken from infant rats (125). In addition, oligodendrocyte cells express FGF receptors, and FGF2 application to cultured cells stimulates proliferation of oligodendrocyte precursor cells (126). FGF2 has also recently been identified as a critical regulator of myelin sheath thickness. Furusho et al. (127) created a line of mutant mice that lacked the FGF receptors 1 and 2, the two receptors to which FGF2 binds. They reported that while mutant mice exhibited normal initiation of myelination in the spinal cord at P4 (as judged by immunoblotting for MBP), by P30 mutant mice exhibited significantly less MBP positive myelin, and reduced overall white matter area, compared to control mice, suggesting a reduction in myelin synthesis. Accordingly, while myelin thickness increased from P15 to 10 months of age (the oldest age tested) in control mice, myelin thickness stalled in mutant mice, who exhibited thinner myelin compared to control mice from PND 30 to 10 months of age. Importantly, the numbers of myelinated and unmyelinated axons was comparable in control and mutant mice at all ages tested, suggesting that FGF2 plays a specific role in signaling for the development of myelin thickness. Hence, it is possible that early life exposure to FGF2, stress, or to stress hormones may help to precociously terminate critical periods in fear learning via accelerating the rate of myelin development in the hippocampus, amygdala, and mPFC.

Along with potentially accelerating structural brakes in plasticity, it is also possible that early life stress/CORT/FGF2 exposure caused an early termination of infantile amnesia, impaired context learning, and erasure-like extinction via a CREB-mediated pathway. For example, many of FGF2’s neurotrophic effects appear to be mediated by phosphorylation of CREB. Sung et al. (128) showed that FGF2 increases hippocampal neuronal differentiation and outgrowth via causing phosphorylation of CREB and CRE-mediated gene transcription. They also demonstrated that FGF2-induced neuronal outgrowth was blocked in cells that contained a dominant negative CREB construct (blocking CREB activation). FGF2 also appears to regulate hippocampal cell proliferation via phosphorylation of CREB (129), and FGF2-induced cell proliferation is blocked by a CREB inhibitor. Cell proliferation was markedly increased in cell cultures that over-expressed CREB, but only if FGF2 was applied to these cultures. In other words, CREB over-expression did not increase cell proliferation by itself, suggesting that FGF2 recruits CREB to increase cell proliferation. In addition, recent research has shown that early exposure to stressors (e.g., maternal-separation) regulates the expression of non-coding RNAs which are mediated by CREB. Specifically, Uchida et al. (130) showed that MS180 from P2 to P14 increased the expression of mir132 in the PFC of P14 mice relative to SR P14 mice. Furthermore, FGF2 has been shown to upregulate mir132 in cultured immature cortical neurons, as well as in cultured astroglial cells (131). As mentioned earlier, alterations in the expression of mir132 have been shown to regulate critical period timing for OD plasticity.

**Figure 2:**
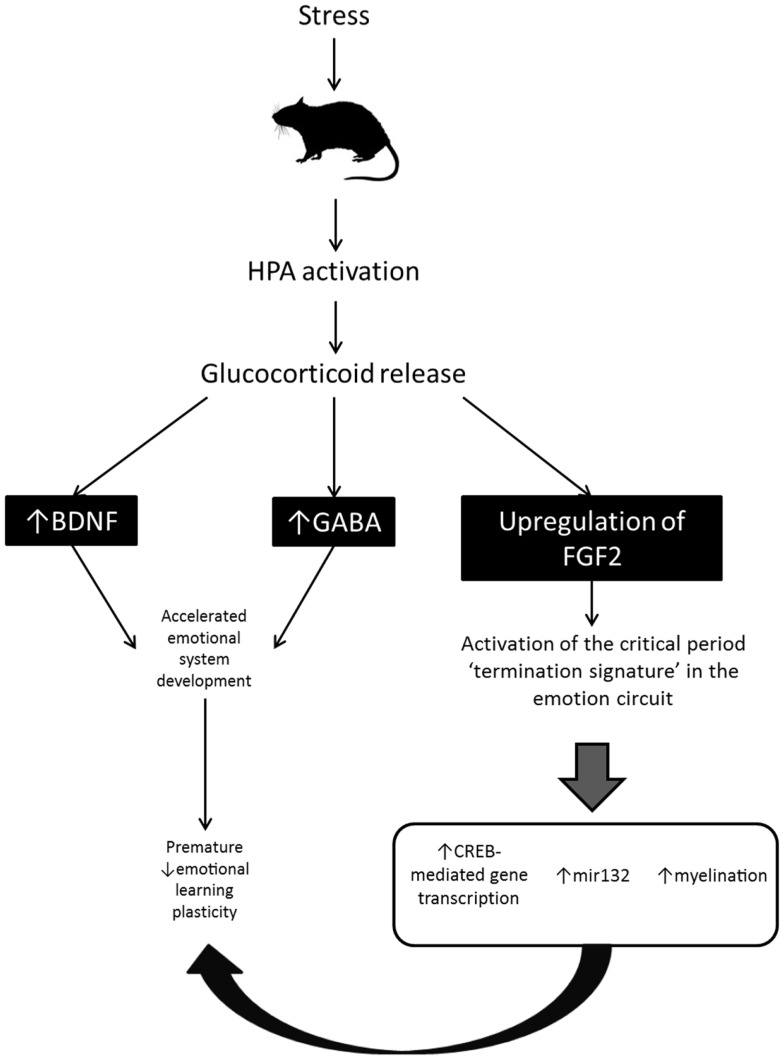
**Proposed mechanism by which chronic stress accelerates the developmental transition between infant and adult-like forms of fear retention and fear learning in rodent models**. Stress-induced activation of the HPA axis results in increased BDNF and GABA, and central upregulation of FGF2. BDNF and GABA stimulate early development of the emotion system and may lead to early opening of the critical period for infantile amnesia and erasure-like extinction. FGF2 upregulation triggers activation of the critical period “termination signature” in the emotion circuit (i.e., activates the cellular and molecular mechanisms known to be involved in critical period timing in sensory systems). Activation of those signals leads to an early termination of fear learning plasticity.

Together the findings just reviewed suggest that early life exposure to FGF2/stress/CORT may regulate the developmental timing of critical periods in fear learning via accelerated maturation of BDNF expression, GABAergic inhibition, myelination, and CREB-mediated gene transcription in those brain regions critical for fear memory and extinction learning in adults – the hippocampus, mPFC, and amygdala (see Figure 2). If this were true, it would support the idea that there may be a common neural signature which guides critical periods of plasticity across the brain. This idea has been raised by previous researchers to explain the finding that the same molecular and cellular signals appear to be involved in a variety of critical periods in different sensory systems (108). However, the idea that the same molecular and cellular signals may regulate critical periods of plasticity for fear learning in subcortical circuits (e.g., the amygdala) has only recently begun to be explored [e.g., (46)].

## Potential for Multigenerational Effects of Stress/FGF2 on Fear Learning

If critical period mechanisms are involved in regulating the opening and closure of fear learning plasticity then a potential implication is that the effects of environmental manipulations on maturation of fear learning might be heritable. Indeed, epidemiological evidence suggests that the effects of stress on mental health can be transmitted across multiple generations. For instance, mothers that were exposed to the September 11 terrorist attacks in New York City during pregnancy and who subsequently developed PTSD were shown to exhibit a suppressed basal cortisol response (132). Interestingly, a similar profile of cortisol suppression was also evident in the infants of those mothers, with that being especially true for infants of mothers that were in the third trimester of pregnancy when the attacks occurred. In addition, high risk phenotypic traits for mental health problems (e.g., behavioral inhibition) have been shown to exhibit a high degree of heritability, which can be attributed to both genetic and environmental factors (133–136). Hence, there is clear epidemiological data suggesting that mental health disorders and the influence of stress on the emergence of those disorders is heritable.

Animal models have been increasingly used to investigate the intergenerational transmission of neurobehavioral alterations after stress (137–140). Several studies in rodents have shown that stress-evoked alterations in parenting style are passed onto offspring, and that these behavioral alterations are often accompanied by neuroendocrine changes (141). In addition, epigenetic modifications to gene transcription caused by early life stress have been shown to persist across the life of the rat and to be passed onto biological offspring (137). Interestingly, more recent studies have demonstrated that actual stress-induced behavioral phenotypes can also be transmitted across generations. For example, maternally separated rats exhibited depressed behaviors as adults, and these same depressive behaviors were also exhibited by their adult offspring and grandchildren, despite those subsequent generations never being exposed to stress (138). Hence, animal research has been useful in modeling the transmission of both neurological as well as behavioral alterations caused by stress. One currently unexplored possibility is that stress-induced alterations to the maturation of fear retention and extinction systems could also be transmitted to subsequent generations. Indeed, some of the mechanisms involved in critical period opening and closure could potentially lead to such a transgenerational profile. Specifically, research has shown that transgenerational effects can be produced by alteration of cytoplasmic RNAs (e.g., miRNA), which can be carried in the sperm and eggs and can epigenetically alter the phenotype of subsequent offspring. Recently it has been proposed that miRNAs may be important in the transmission of environmentally induced phenotypic changes across generations because some RNAs can survive degradation during embryogenesis and have been shown to regulate offspring phenotype [e.g., (142)]. The evidence for this comes from experiments which show that injection of a miRNA critical for brain development [mir124; (119, 143)] directly into cell embryos resulted in offspring which exhibited a much faster growth rate (increased by 30%) than non-injected offspring (144). Importantly, this “giant” phenotype was transmitted across multiple generations via alterations of mir124 in the spermatozoa. Hence, changes in the expression levels of certain miRNAs can be incorporated into the germ-line of animals and produce a transgenerational phenotype. As mentioned earlier, a recent study showed that a miRNA important for inhibiting OD plasticity in the visual cortex (mir132) and the miRNA which produced a transgenerational “giant” phenotype (mir124) was upregulated in the mPFC of P14 mice following maternal-separation (130). Further, mir132 is upregulated by FGF2 (131). Hence, it is possible that the expression of these miRNAs may regulate critical period closure in fear learning systems and that stress/FGF2-induced alterations in these miRNAs could be heritable. Such hypotheses will need to be investigated in future studies.

## Bridging the Gap between Basic and Clinical Work: Clinical Implications and Potential Translation of Stress/FGF2-Induced Acceleration of Emotional Development in Animal Models

The fact that infantile amnesia and relapse-resistant extinction are regulated by stress, FGF2, and potentially other early life events is highly relevant for clinical researchers working on understanding and treating mental health disorders across the lifespan. Early life stress is one of the greatest contributing risk factors for mental health problems across all life stages (145), relating not only to risk for mental health disorders but also to transdiagnostic features common of many psychological disorders [e.g., increased emotional reactivity; (146, 147)]. Further, early adversity and abuse has been shown in human populations to interact with specific genetic polymorphisms to predict adult major depressive disorder and PTSD (148, 149). However, the developmental trajectories which are altered by such gene × environment interactions remain elusive. The body of research reviewed in this paper suggests that early emerging changes in fear learning and extinction resulting from stress may be one outcome which could affect emotional responding across the lifespan and which might interact with genetics to produce stable phenotypes of risk for mental health disorders. For example, it is possible that stress exposure during a critical period of development early in life paired with a later experienced trauma might lead to a phenotype of treatment-resistant PTSD in genetically predisposed individuals via a pathway of altered development of the fear extinction system; such a possibility should be examined in future studies.

The possibility that infantile amnesia and relapse-resistant extinction may represent critical period plasticity in fear learning also has significant clinical implications, especially when considering potential pharmacological treatments for mental health disorders. As discussed earlier, the involvement of critical period molecular signals in terminating fear learning plasticity opens up a possible mechanism via which the effects of stress/FGF2 exposure might increase vulnerability for mental health problems across multiple generations. In addition, they also suggest several novel mechanisms via which anxiety disorders and other mental health problems might be treated. Specifically, if critical periods of emotional learning could be reopened in adulthood (or at any point after they have closed) it may help treat the root of many anxiety disorders (i.e., persistent expression of fear and relapse after extinction). In other words, it is possible that anxious individuals might be treated with pharmacological adjuncts to reopen infant-like forgetting and relapse-resistant extinction, which could then be combined with therapy to improve treatment efficacy. Indeed, there have been three recent studies which suggest that the critical period of erasure-like (relapse-resistant) extinction can be reopened in juvenile and adult rats. The first evidence that relapse-resistant/erasure-like extinction could be reactivated in adult rats came from Gogolla et al. (46). In those studies appearance of PNNs around GABAergic amygdala interneurons was correlated with the natural transition from relapse-resistant extinction in infant mice to relapse-prone extinction in juvenile mice. That is, at the same time that rats began to exhibit relapse behaviors after extinction there was a significant increase in the number of PNNs in the amygdala. To examine whether the formation of the PNNs was sufficient to cause the transition into adult-like extinction Gogolla et al. degraded amygdala PNNs with chABC in adult mice before conditioning. The treatment with chABC significantly reduced the number of PNNs in the adult amygdala and also reduced the expression of relapse behaviors after extinction (i.e., the chABC-treated adults did not show renewal or spontaneous recovery of extinguished fear). Hence, it appears that the infant profile of extinction learning could be reactivated in adulthood by removal of one of the structural brakes on plasticity – PNNs.

Another line of evidence that “erasure-like” extinction can potentially be activated in adult rats comes from recent work on the impact of acute, exogenous FGF2 on extinction of conditioned fear (150–153). Those studies demonstrated that systemic or intra-amygdala infusion of FGF2 not only enhanced extinction in juvenile and adult rats, but it also significantly reduced renewal and reinstatement, even when vehicle-treated rats were given four times the amount of extinction training to match extinction strength between vehicle- and FGF2-treated groups. In other words, when treated with FGF2, adult rats exhibit the behavioral qualities of infant-like (erasure-like) extinction. The neurobiological mechanisms by which FGF2 causes infant-like extinction are unknown. Nevertheless, similar to findings in the visual system, it appears that adult rats retain the capacity for infant-like extinction and that this form of plasticity can be reactivated rapidly under conditions which favor that plasticity.

In order to investigate the possibility that extinction combined with FGF2 leads to an erasure of the original fear memory, Graham and Richardson (152) exploited recent findings regarding re-extinction, which refers to the process of relearning extinction following reacquisition of fear to an extinguished cue. Converging evidence strongly suggests that whereas initial extinction in adult rats is impaired by NMDAr antagonists, re-extinction is not impaired by NMDAr antagonists (154–157). This suggests that relearning to extinguish fear does not depend on NMDAr activity. However, Graham and Richardson (152) found that when rats were systemically injected with FGF2 immediately after extinction training, then retrained to fear the extinguished CS, and then re-extinguished following treatment with an NMDAr antagonist, FGF2-treated rats exhibited impaired re-extinction retention. In contrast, rats that were extinguished with vehicle and then re-extinguished following treatment with an NMDAr antagonist did not exhibit any impairment in re-extinction retention. That is, during re-extinction FGF2-treated rats “behaved” as if the CS was being extinguished for the first time. Interestingly, similar results have been obtained for juvenile rats that are extinguished to a CS at PND 16 (during the “erasure-like extinction” period of development), and then retrained and re-extinguished to the same CS later in development. In this instance, re-extinction is also NMDAr-dependent (158). Together, these findings suggest that FGF2 treatment, when combined with extinction training, may reactivate the “erasure-like” fear extinction observed in infant rats.

The third study to attempt to reactive infant-like plasticity in rodents during extinction learning was performed by Karpova et al. (159). In that study adult mice were chronically exposed to the antidepressant fluoxetine in their drinking water either before or after fear conditioning and during extinction and test. They showed that the fluoxetine-exposed mice behaved like infant mice in past studies (46), showing less post-extinction relapse than the vehicle-treated mice. In addition, fluoxetine treatment also resulted in a lower proportion of PNNs in the BLA, suggesting that the effect of fluoxetine on relapse behaviors after extinction may have occurred through facilitating the removal of structural brakes on plasticity (PNNs). Interestingly, combining antidepressant treatments like fluoxetine with exposure therapy in humans has often yielded better results than either treatment alone (160). The study by Karpova et al. (159) suggests that fluoxetine-induced reactivation of the critical period for erasure-like extinction might underlie those clinical findings.

## Conclusion

The findings regarding accelerated development of fear learning by stress/CORT/FGF2 are theoretically relevant because they demonstrate that the rate at which particular forms of learning and memory mature across the lifespan can be influenced by a range of early life experiences. Until recently, no one had examined how early experiences affected fear retention and extinction development, despite these forms of emotional learning being critically involved in the pathogenesis and treatment of mental health problems. The studies reviewed here show that the timing of the maturation of fear learning is not set in stone but can be dynamically regulated by early experience. In addition, these findings are clinically relevant because early life adversity is a common feature in persons with psychopathology [e.g., (161, 162)], and fear retention and extinction in rats are important pre-clinical models of anxiety problems in humans (10, 163, 164). Although many theories have suggested that early experiences are critical for the emergence of anxiety and other mental health problems in humans (165–168), no studies, until very recently, had examined how fear retention and extinction are impacted by different early experiences in infant rodents. In addition, within the human literature, there are reports of individual differences in the processes of fear retention and extinction which may underlie subsequent vulnerability to develop anxiety problems [e.g., (169, 170)], yet there is little information on what factors might influence those differences or the molecular mechanisms which might underlie them.

While the findings regarding environmental alteration of the maturation of fear learning systems are novel, at this stage there are no definitive answers about what molecular and cellular mechanisms drive the normal development of these emotion systems, nor the accelerated transition produced by stress/CORT/FGF2. However, the fact that all three manipulations have a similar effect on emotion system development, that stress/CORT regulate FGF2, and that stress/CORT and FGF2 appear to regulate some of the signals involved in critical periods of plasticity in sensory systems hints at a potential mechanism for transitions in fear learning. Specifically, we have suggested that the expression of infantile amnesia and relapse-resistant extinction in infancy may represent critical period plasticity and propose a model in which early environments that alter the age at which the developmental transitions occur (e.g., stress) might function through an HPA/FGF2-dependent activation of “critical period signals,” in turn leading to an early termination in emotional plasticity (see Figure 2 for a graphical depiction of this model). The proposed model, although speculative, does suggest some potential avenues for future research. Specifically, if the principles guiding critical period plasticity in sensory systems can also be generalized to emotion learning, it should be possible to manipulate the timing of infantile amnesia and erasure-like extinction via alteration of any of the signaling pathways involved in critical period plasticity in sensory systems. Also, interfering with any of the signaling pathways involved in critical period plasticity should change the effect of stress on fear retention and extinction learning in infant rats. One possibility, for example, might be to chronically suppress levels of BDNF while rats are experiencing maternal-separation to determine whether accelerated emergence of adult-like fear retention and extinction still occurs. All these possibilities would have important outcomes both theoretically, in understanding the guiding principles of critical period plasticity, as well as clinically, in understanding how particular experiences might impact emotional development across the life span. Although these speculations require further examination, the reviewed literature is clearly developing a foundation for examining the experience-dependent modulation of critical period opening and closure in emotional systems, an area with significant implications for our understanding and treatment of anxiety disorders (e.g., PTSD).

## Conflict of Interest Statement

The authors declare that the research was conducted in the absence of any commercial or financial relationships that could be construed as a potential conflict of interest.
